# Bidirectional Causality between Spreading COVID-19 and Individual Mobilisation with Consumption Motives across Prefectural Borders in Japan

**DOI:** 10.3390/ijerph19159070

**Published:** 2022-07-25

**Authors:** Yasuhiro Kawano, Ryusuke Matsumoto, Eishi Motomura, Takashi Shiroyama, Motohiro Okada

**Affiliations:** Department of Neuropsychiatry, Division of Neuroscience, Graduate School of Medicine, Mie University, Tsu 514-8507, Japan; y-kawano@clin.medic.mie-u.ac.jp (Y.K.); matsumoto-r@clin.medic.mie-u.ac.jp (R.M.); motomura@clin.medic.mie-u.ac.jp (E.M.); takashi@clin.medic.mie-u.ac.jp (T.S.)

**Keywords:** COVID-19, Japan, prefecture, epicentre, individual mobilisation, consumption

## Abstract

A combination of pharmaceutical and non-pharmaceutical interventions as well as social restrictions has been recommended to prevent the spread of coronavirus disease 2019 (COVID-19). Therefore, social contact surveys play an essential role as the basis for more effective measures. This study attempts to explore the fundamental basis of the expansion of COVID-19. Temporal bidirectional causalities between the numbers of newly confirmed COVID-19 cases (NCCC) and individual mobilisations with consumption motives across prefecture borders in three metropolitan regions in Japan were analysed using vector autoregression models. Mobilisation with consumption in pubs from Kanto to Tokai contributed to the spread of COVID-19 in both regions. Meanwhile, causal mobilisation with consumption motives in Kansai also contributed to the expansion of COVID-19; however, the pattern was dependent on the industrial characteristics of each prefecture in Kansai. Furthermore, the number of pub visitors in Kanto immediately decreased when NCCC increased in Kanto. In contrast, the causal mobilisations for the expansion of COVID-19 in the Tokai and Kansai regions were unaffected by the increasing NCCC. These findings partially proved the validity of the conventional governmental measures to suppress pub visitors across prefectural borders. Nevertheless, the individual causal mobilisations with consumption motives that contributed to the increasing COVID-19 cases are not identical nationwide, and thus, regional characteristics should be considered when devising preventive strategies.

## 1. Introduction

The ongoing coronavirus disease 2019 (COVID-19) was first detected in Wuhan, in the Hubei province of China, on 8 December 2019 [1]. Due to the highly contagious and pathogenic nature of the disease [2], it was declared a public health emergency of international concern on 30 January 2020 [3], and was also declared a pandemic by the World Health Organization on 11 March 2020 [4]. As of 1 June 2022, more than 540 million confirmed cases of COVID-19 were recorded, of which approximately 6.3 million were fatalities [4]. The importance of interpreting the propagation dynamics of infectious diseases through social contact among individuals was established from the outbreaks of both Ebola and influenza [5,6]. Based on the previous findings, social contact surveys also provided several fundamental insights into the importance of COVID-19 prevention [7].

**Figure 1:**
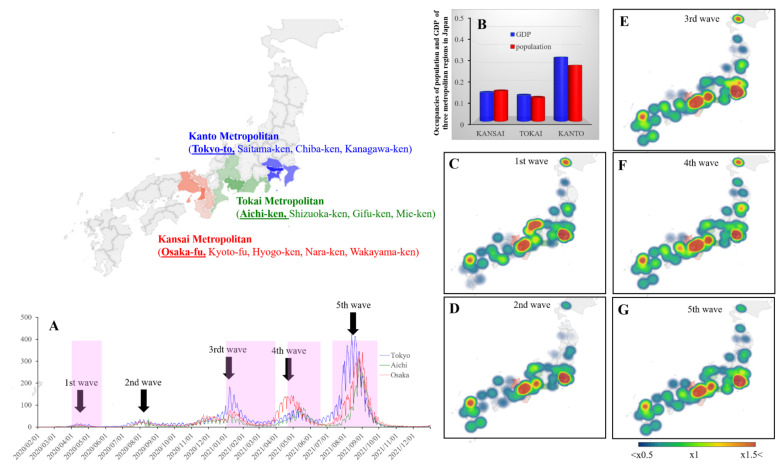
Geographical features of three (Kanto, Tokai, and Kansai) metropolitan regions and the five waves of the COVID-19 pandemic. (**A**) indicates the time series transition of the number of confirmed COVID-19 cases in Kanto (blue), Tokai (green) and Kansai (red) metropolitan regions (per 1,000,000 population). (**B**) represents the occupancies of population and GDP of the three metropolitan regions (%). (**C**–**G**) indicate the relative cumulative confirmed COVID-19 cases in each pandemic wave per national average (%).

The combination of clinical interventions (vaccines and medication), non-pharmaceutical interventions (mask wearing and hand sanitiser use), and social restrictions (border control, international voyage restrictions, social distancing requirements, stay-at-home orders, and/or lockdowns) have been recommended to prevent the domestic spread of the virus, particularly for airborne pathogens, such as SARS-CoV-2 [8,9,10]. Indeed, at the early stages of the COVID-19 pandemic, several Asian countries were able to contain the spread of SARS-CoV-2 through travel bans and restrictions [11]. However, despite these efforts, once SARS-CoV-2 invaded these countries, the infection spread at an unprecedented rate, partly owing to asymptomatic and/or presymptomatic carriers [12]. As a countermeasure against the domestic spread, severe public health, and social restrictions have been required [10]. Nevertheless, after a relaxation of social restrictions to the seemingly decreased prevalence of COVID-19, a surge of cases has been observed due to new COVID-19 variants (Figure 1) [10]. These repeated restrictions have deteriorated the socioeconomic and psychosocial conditions of individuals [13,14]. Therefore, exploring other social restrictions (suppression of high-risk behaviours) that are efficient in preventing COVID-19 with less associated socioeconomic and/or sociopsychological deteriorations is necessary.

The propagation of the viral infection is not dependent on two-dimensional geographical spreading, but rather on spatial heterogeneity (Figure 1) [15,16]. It has been considered that the spatial heterogeneity of the propagation of viral infections has been regulated by various regional factors, such as population density, prevention policy, medical level, and transportation networks [15,17,18]. The spatial dependencies between cities play important roles in the spatial heterogeneity of the epidemic distribution [15,16]. In the analysis of the SARS-CoV-2 transmission through the transportation network during the first pandemic in China (until 29 February 2020), with the epicentre in Wuhan, the distributions of cities suffering from a serious epidemic showed a punctate pattern that overlapped with the air transportation networks and banded patterns dependent on the high coefficients of rail passenger density [15]. Notably, the transportation networks associated with the spread of SARS-CoV-2 were observed in direct transportation routes to Wuhan and also in the regional hub cities without direct transportation routes [15]. These findings suggest that the combination of restrictions between direct routes to nationwide and regional hub-epicentres is more efficient in preventing the spread of SARS-CoV-2 rather than non-specific restrictions across the whole country.

However, restrictions of the transportation network alone were focused on comprehensively blocking human and material flows, possibly resulting in a severe deterioration of individuals’ socioeconomic and sociopsychological conditions. Individuals move/ambulate for specific purposes, such as work, education, consumption, or recreation. In Japan, the first “state of emergency” (a stay-at-home order targeted at reducing individual mobilisation at night-time) could not decrease the night-time population in the epicentre regions [19]. Notably, despite the severe governmental social restriction measures under the state of emergency, the fifth wave of the COVID-19 pandemic, due to the Delta variant, occurred, and the contribution of the mobilising population in Tokyo to the reproduction number was inconsistent [20]. These previous findings indicated that comprehensive social restrictions could not sufficiently prevent the spread of SARS-CoV-2. Therefore, to sufficiently prevent the spread of COVID-19 without socioeconomic and/or sociopsychological deteriorations, we must identify the specific social contact patterns which play crucial roles in the spread of SARS-CoV-2. It is established that the interpretation of the social contact surveys is complicated compared with a geographic analysis of COVID-19 since social contact behaviours were also drastically affected by personal factors due to the progression of the pandemic itself and the governmental social restricting measures [19,20,21,22].

Identifying the causal motives of individual mobilisations, which contribute to the regional expansion of the COVID-19 pandemic, can help to develop reasonable measures for efficient mitigation of the ongoing COVID-19 pandemic and a future new pandemic without socioeconomic and/or sociopsychological deteriorations. Additionally, intensive restriction measures on the individual mobilisations which are identified as the major pandemic propagating factors, and a concentrated government assurance against restrictions (if needed), possibly suppresses not only socioeconomic deterioration but also sociopsychological deterioration as much as possible [23,24,25]. Based on these socioeconomic/sociopsychological situations, the present study is based on two major research questions: whether there are causal motives of individual mobilisations, which contribute to the spread of SARS-CoV-2, and whether these individual mobilisations are affected by the spread of COVID-19. Therefore, the present study attempted to determine the temporal bidirectional causalities among the numbers of newly confirmed COVID-19 cases (NCCC) and individual mobilisations with consumption motives within prefectures and across prefecture borders using personal consumption databases.

## 2. Materials and Methods

### 2.1. Data Sources

The daily prefectural numbers of NCCC were obtained from the Database of the National Institute of Infectious Diseases (NIID) [26] and the Sapporo Medical University School of Medicine [27]. Meanwhile, data on the prefectural populations were obtained from the Regional Statistics Database (RSD) of the System of Social and Demographic Statistics of the Statistics Bureau of the Ministry of Internal Affairs and Communications (SBMIAC) [28].

The personal consumption data were purchased from “JCB Consumption NOW” (Nowcast, Tokyo, Japan) [29]. The number of card members and the annual transaction volume of JCB was 141 million people and 305.5 billion USD, respectively (in 2020) [30]. The consumption statistics from JCB Consumption NOW provide the JCB credit card transaction data for actual purchases (except the members who registered not to authorise the use of their data), which records information on the buyer side, such as how much a consumer spent at which store on which day [29]. Importantly, any information in JCB Consumption NOW is provided on the premise that measures are taken to maintain confidentiality by making it impossible to identify individuals so that the day-to-day purchasing behaviour of individuals cannot be traced. In this study, we adopted the From-To data in the JCB Consumption NOW database, which provides bimonthly numbers of consumers of hotels, business hotels, amusement places, pubs, department stores, and shopping malls, disaggregated by the individual information (from their home location to their destination prefectures with consumption).

The consumption amount, as per the governmental consumption survey “Family Income and Expenditure Survey”, which is a questionnaire survey of 9000 households, is 30 billion JPY (providing monthly data), whereas the amount as per JCB Consumption NOW, which involves the actual purchase statistics, is 4 trillion JPY (providing bimonthly data), [29,31]. Therefore, JCB Consumption NOW provides the largest and most relevant consumer statistics available in Japan. Indeed, due to this usefulness/effectiveness, JCB consumption NOW has been adopted by the RESAS database “Regional Economy and Society Analysing System” and V-RESAS database “Vital Signs of Economy Regional Economy and Society Analysing System”, published by the Cabinet Office [32,33].

### 2.2. Target Regions and Period

In Japan, serious and widespread COVID-19 was observed in five metropolitan regions, which include prefectures with populations of more than 5 million (Figure 1) [34,35]. Three of the five metropolitan regions have urban functions spanning across multiple prefectures: Kanto (Tokyo-to, Saitama-ken, Chiba-ken and Kanagawa-ken), Tokai (Aichi-ken, Gifu-ken, Shizuoka-ken and Mie-ken), and Kansai (Osaka-fu, Kyoto-fu, Hyogo-ken, Nara-ken and Wakayama-ken) metropolitan regions (Figure 1). These three metropolitan regions boast more than 50% of the population and GDP in Japan (Figure 1). Tokyo-to (Tokyo special wards), Aichi-ken (Nagoya City), and Osaka-fu (Osaka City) play a central role in urban functions in the Kanto, Tokai, and Kansai metropolitan regions, respectively. Until December 2021, Japan had experienced five pandemic waves, which were expanding from Tokyo or Osaka (as epicentres) (Figure 1). Based on these backgrounds, these three metropolitan regions were adopted as the target regions in this study.

The study began after 16 March 2020, since the newly confirmed COVID-19 cases were reported in all 13 target prefectures during the second half of March 2020. Serial intervals of the SARS-CoV-2 Omicron variants and others were reported to be 3.1 and 4.0 days, respectively [36,37]. The sixth wave of the pandemic was due to the Omicron variant. For this reason, the study ended in December 2021 to eliminate the impact of Omicron variants. Therefore, the study period was set between 16 March 2020 and 31 December 2021.

### 2.3. Statistical Analysis

The main interest of this study was the identification of the consumption motives in individual cross-prefectural border mobilisations that contributed to the COVID-19 expansion. The temporal bidirectional causalities among the prefectural NCCC and the visitor numbers (domestic and from out-of-prefecture) for department stores, shopping malls, hotels, business hotels, amusement places, and pubs were analysed by a vector autoregression with Granger causality models using gretl v2021d (accessed on 28 December 2021). When the assumption of Granger causality was violated (*p* < 0.05), the forecast variance decomposition, sensitivity analysis, and impulse response analysis were also conducted. The lag number was evaluated using the Akaike information criterion. Especially, the dependent variable was set as the first shock to avoid false positives, since the first shock affects the impulse responses predominantly among the variables in the vector autoregression model. The regression model considering three vectors of time series data with p logs was:

Xt = ν1 + ρ1Ct + α11Xt-1 +…+α1pXt-p + β11Yt-1 +…+β1pYt-p + γ11Zt-1 +…+ γ 1pZt-p + υ1

Yt = ν2 + ρ2Ct + α21Xt-1 +…+α2pXt-p + β21Yt-1 +…+β2pYt-p + γ21Zt-1 +…+ γ 2pZt-p + υ2

Zt = ν3 + ρ3Ct + α31Xt-1 +…+α3pXt-p + β31Yt-1 +…+β3pYt-p + γ31Zt-1 +…+ γ 3pZt-p + υ3

ν: the intercept; ρ: the coefficient of exogenous variable C; α: the coefficient of the lagged terms of X; β: the coefficient of the lagged terms of Y; γ: the coefficient of the lagged terms of Z; υ: assumed to be white noise.

## 3. Results

### 3.1. Individual Mobilisations between the Centre Prefectures in Three Metropolitan Regions

**Figure 2:**
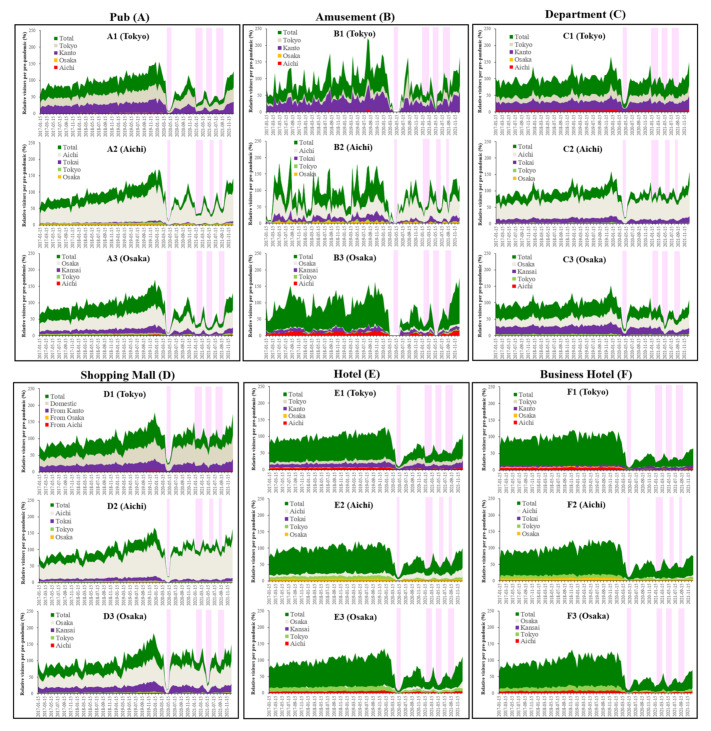
Fluctuations of bimonthly visitor numbers of pubs (**A**), amusement places (**B**), department stores (**C**), shopping malls (**D**), hotels (**E**), and business hotels (**F**) in Tokyo (1), Aichi (2), and Osaka (3) between January 2017 and December 2021 in Japan. Ordinates indicate the relative bimonthly visitor numbers per the average of visitor numbers before the pandemic (January 2017–December 2019) (%). Light red columns indicate the periods of governmental social restriction, “State of emergency” to prevent the COVID-19 epidemic.

The number of visitors to department stores, shopping malls, pubs, amusement places, hotels, and business hotels in Tokyo, Aichi, and Osaka decreased during the COVID-19 pandemic compared to before the pandemic (Figure 2).

The majority of the visitors to pubs, department stores, and shopping malls were domestic individuals, but those to hotels and business hotels were predominantly individuals from out-of-metropolitan regions (Figure 2). The domestic visitors to amusement places in Tokyo, Aichi, and Osaka were 45%, 55%, and 20%, respectively (Figure 2). Visitors to all six sites from the other prefectures were lesser than domestic visitors (Figure 2). Notably, similar to the previous findings regarding the night-time population in Tokyo [19], the onset period of decreasing visitors to all six sites in the three metropolitan regions was synchronised with the onset of increasing NCCC in these prefectures and was prior to the announcement of the governmental restriction measures “state of emergency (stay-at-home order)” (Figure 1 and Figure 2).

### 3.2. Inter-Metropolitan Regions Causalities

**Figure 3:**
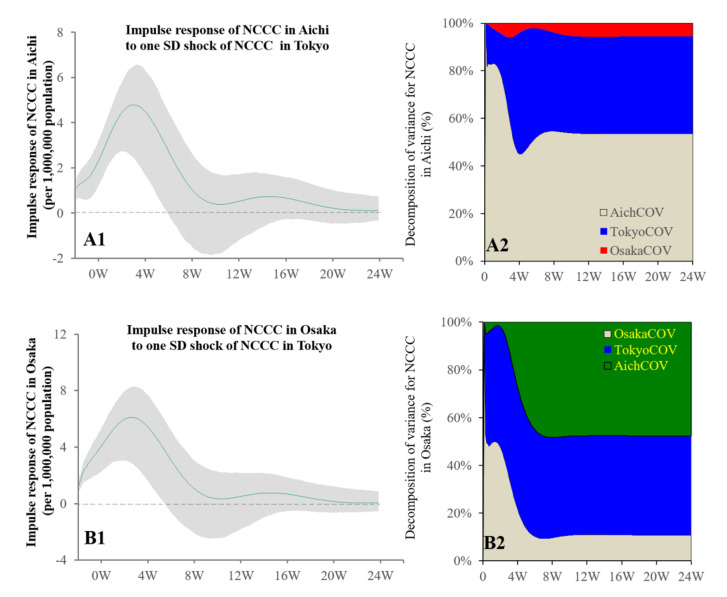
Temporal bidirectional causalities of daily NCCC among centre prefectures of three (Kanto, Tokai, and Kansai) metropolitan regions. Left side panels (**A1**,**B1**) indicate the impulse responses of NCCC in Aichi and Osaka to increasing NCCC in Tokyo, respectively. Green lines and grey regions indicate the mean ± 95% confidence interval (CI) of responses. Right side panels (**A2**,**B2**) indicate the decomposition of variances of three metropolitan regions for NCCC in Aichi and Osaka, respectively.

Granger causality could detect the causality from the NCCC (daily) in Tokyo to the NCCC in Aichi and Osaka (daily), whereas neither those in Aichi nor Osaka to the NCCC in Tokyo were detected. The bidirectional causality of the NCCC values between Aichi and Osaka also could not be detected. The impacts of the NCCC in Tokyo were positively related (increasing) to those in Aichi and Osaka (Figure 3).

**Figure 4:**
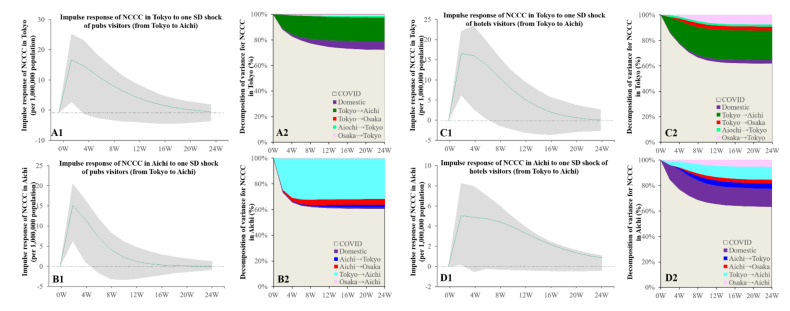
Temporal bidirectional causalities of bimonthly visitor numbers of pubs and hotels to bimonthly NCCC among centre prefectures of three (Kanto, Tokai, and Kansai) metropolitan regions. Left side panels (**A1**,**B1**,**C1**,**D1**) indicate the impulse responses of NCCC to increasing visitor numbers. Green lines and grey regions indicate the mean ± 95% CI of responses. Right side panels (**A2**,**B2**,**C2**,**D2**) indicate the decomposition of variances of NCCC in three metropolitan regions (%).

Granger causality detected the causalities from the bimonthly visitor numbers (from Tokyo to Aichi) to the NCCC values in both Aichi and Tokyo but could not detect the causality from the visitor numbers (from Aichi or Osaka to Tokyo) to the NCCC in the three prefectures (Figure 4). The increasing individual mobilisations with consumption in pubs (from Tokyo to Aichi) was related to the increase in the NCCC in both Tokyo and Aichi (Figure 4). The increasing number of hotel visitors (from Tokyo to Aichi) was also related to the increasing NCCC in Tokyo and Aichi (Figure 4).

Granger causality detected only the causality from the bimonthly NCCC to the bimonthly mobilisation in visitor numbers of business hotels (Supplementary Figure S1). Only the increasing NCCC in Tokyo was related to the decreasing number of individual mobilisations staying in business hotels (from Aichi and Osaka to Tokyo, as well as from Tokyo to Aichi) (Supplementary Figure S1). Contrary to the NCCC in Tokyo, neither the NCCCs in Aichi nor Osaka were related to any individual mobilisations among the three metropolitan regions (Supplementary Figure S1).

### 3.3. Intra-Relations in Kanto Metropolitan Region

**Figure 5:**
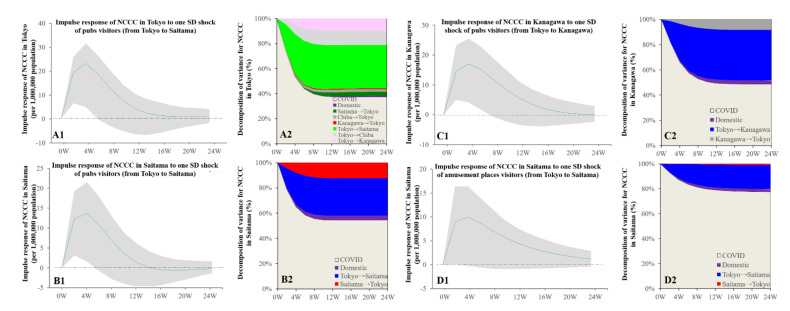
Temporal bidirectional causalities of bimonthly numbers of pub visitors to bimonthly NCCC among prefectures in the Kanto metropolitan region. Left side panels (**A1**,**B1**,**C1**,**D1**) indicate the impulse responses of NCCC to increasing pub visitors. Green lines and grey regions indicate the mean ± 95% CI of responses. Right side panels (**A2**,**B2**,**C2**,**D2**) indicate the decomposition of variances for NCCC of the prefecture in the Kanto metropolitan region (%).

Granger causality detected the causality of the NCCC in the Kanto metropolitan region from the bimonthly visitor numbers. The increasing individual mobilisations with consumption in pubs (from Tokyo to Saitama) was related to the increasing NCCC in both Tokyo and Saitama (Figure 5). The increasing pub visitors (from Tokyo to Kanagawa) were also related to the increasing NCCC in Kanagawa (Figure 5). The increasing number of visitors to amusement places (from Tokyo to Saitama) was related to the increasing NCCC in Saitama (Figure 5).

Granger causality could detect the causality of the bimonthly NCCC in Tokyo but not in other prefectures in the Kanto metropolitan region for bimonthly individual mobilisations. The increasing NCCC in Tokyo was related to the decrease in pub visitors (from Tokyo to all three other prefectures; from Saitama and Chiba to Tokyo; and domestic within Tokyo) (Supplementary Figure S2). Moreover, the increasing NCCC in Tokyo was related to the decrease in individual mobilisations to stay at hotels (from Tokyo to Saitama and Kanagawa), as well as being related to a decrease in hotel visitors in Tokyo from all prefectures (Tokyo domestic, Saitama, Chiba, and Kanagawa) (Supplementary Figure S3). Furthermore, the increasing NCCC in Tokyo was related to the decrease in shopping mall visitors from Tokyo to Kanagawa as well as from all prefectures to Tokyo (Supplementary Figure S4).

### 3.4. Intra-Relations in Tokai Metropolitan Region

**Figure 6:**
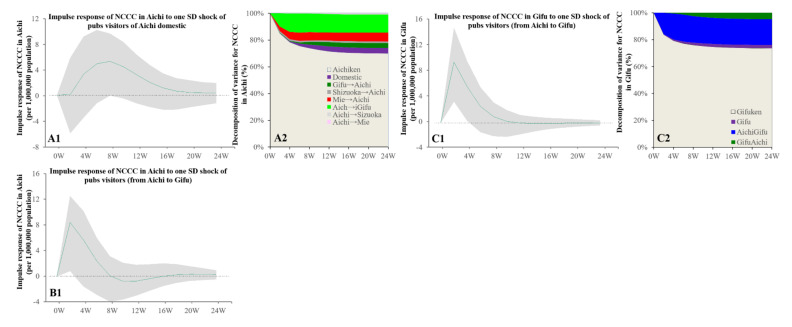
Temporal bidirectional causalities of bimonthly numbers of pub visitors to bimonthly NCCC among prefectures in the Tokai metropolitan region. Left side panels (**A1**,**B1**,**C1**) indicate the impulse responses of NCCC to increasing pub visitors. Green lines and grey regions indicate the mean ± 95% CI of responses. Right side panels (**A2**,**C2**) indicate the decomposition of variances for NCCC of the prefecture in the Tokai metropolitan region (%).

**Figure 7:**
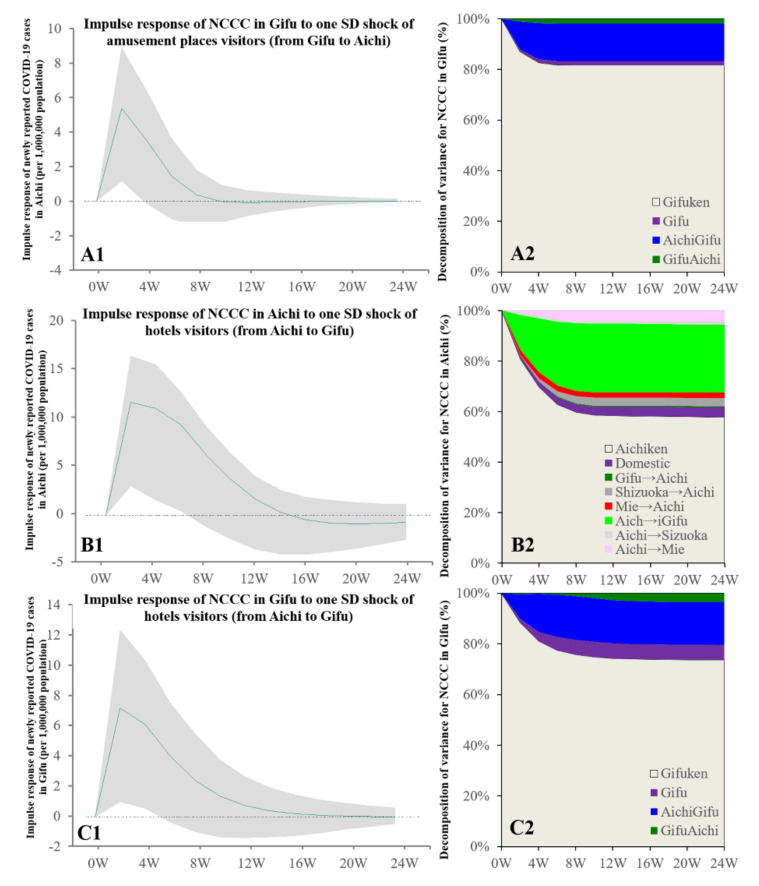
Temporal bidirectional causalities of bimonthly visitor numbers of amusement places and hotels to bimonthly NCCC among prefectures in the Tokai metropolitan region. Left side panels (**A1**,**B1**,**C1**) indicate the impulse responses of NCCC to the increasing number of visitors to amusement places and hotels. Green lines and grey regions indicate the mean ± 95% CI of responses. Right side panels (**A2**,**B2**,**C2**) indicate the decomposition of variances for NCCC of the prefecture in the Tokai metropolitan region (%).

Granger causality detected the causality from the individual mobilisations with consumption motives to the NCCC between Aichi and Gifu, but the causalities among Aichi, Shizuoka, and Mie were not detected. The increasing individual mobilisation with consumption at pubs at domestic Aichi and from Aichi to Gifu was related to the increasing NCCC in both Aichi and Gifu (Figure 6). The increase in visitors (from Gifu to Aichi) to amusement places was related to the increasing NCCC in Gifu (Figure 7). The increase in hotel visitors from Aichi to Gifu was related to the increasing NCCC in both prefectures, similar to the individual mobilisation with consumption in pubs (Figure 7).

Contrary to the Kanto metropolitan region, Granger causality could not detect the causality from the bimonthly NCCC in Aichi to the bimonthly visitors in the Tokai metropolitan region. The increasing NCCC in Gifu was related to the decrease in visitors to amusement places (from Gifu to Aichi), business hotels (from Aichi to Gifu), and domestic mobilisations at business hotels in Gifu (Supplementary Figures S5 and S6). The increasing NCCC in Shizuoka was related to the decrease in visitors to amusement places (from Shizuoka to Aichi), business hotels (Shizuoka domestic, and from Aichi to Shizuoka), pubs (Shizuoka domestic), and hotels (Shizuoka domestic) (Supplementary Figures S5 and S6). The increasing NCCC in Mie was related to the decreasing number of visitors to amusement places (Mie domestic, from Mie to Aichi, and vice versa), business hotels (from Aichi to Mie), and hotels (Mie domestic, and from Aichi to Mie) (Supplementary Figures S5 and S6).

### 3.5. Intra-Relations in Kansai Metropolitan Region

**Figure 8:**
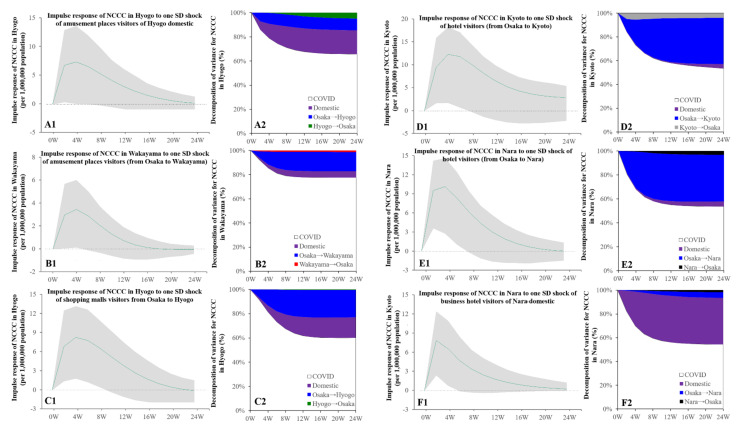
Temporal bidirectional causalities of bimonthly visitor numbers of amusement places, shopping malls, hotels, and business hotels to bimonthly NCCC among prefectures in the Kansai metropolitan region. Left side panels (**A1**,**B1**,**C1**,**D1**,**E1**,**F1**) indicate the impulse responses of NCCC to increasing visitors to amusement places and shopping malls. Green lines and grey regions indicate the mean ± 95% CI of the responses. Right side panels (**A2**,**B2**,**C2**,**D2**,**E2**,**F2**) indicate the decomposition of variances for NCCC of the prefecture in the Kansai metropolitan region (%).

Granger causality detected the causality from the bimonthly individual mobilisations (from Osaka to other prefectures) to the NCCC in the other four prefectures in the Kansai metropolitan region (Kyoto, Hyogo, Nara, and Wakayama); however, there were no causalities from individual mobilisation (from other prefectures to Osaka) to the NCCC in Osaka. The increasing number of hotel visitors from Osaka to Kyoto and Nara was related to the increasing NCCC in Kyoto and Nara, respectively (Figure 8). The increasing number of amusement place visitors from Osaka to Wakayama was related to the increasing NCCC in Wakayama (Figure 8). The increase in shopping mall visitors from Osaka, as well as domestic visits to amusement places in Hyogo, were related to the increasing NCCC in Hyogo (Figure 8). The increase in domestic visits to business hotels in Nara was related to the increasing NCCC in Nara (Figure 8).

Granger causality could not detect the causality of the bimonthly NCCC in Osaka from the bimonthly visitors in the Kansai metropolitan region. The increasing NCCC in Kyoto was related to the decreasing number of pub and business hotel visitors from Kyoto to Osaka (Supplementary Figure S7). The increasing NCCC in Nara was related to the decrease in pub visitors from Nara to Osaka (Supplementary Figure S7). The increasing NCCC in Hyogo was related to the decrease in hotel visitors from Osaka to Hyogo (Supplementary Figure S7), as well as a decrease in domestic visitors to pubs, hotels, and business hotels in Hyogo (Supplementary Figure S7).

### 3.6. COVID-19 Spreading and Alcohol Consumption

**Figure 9:**
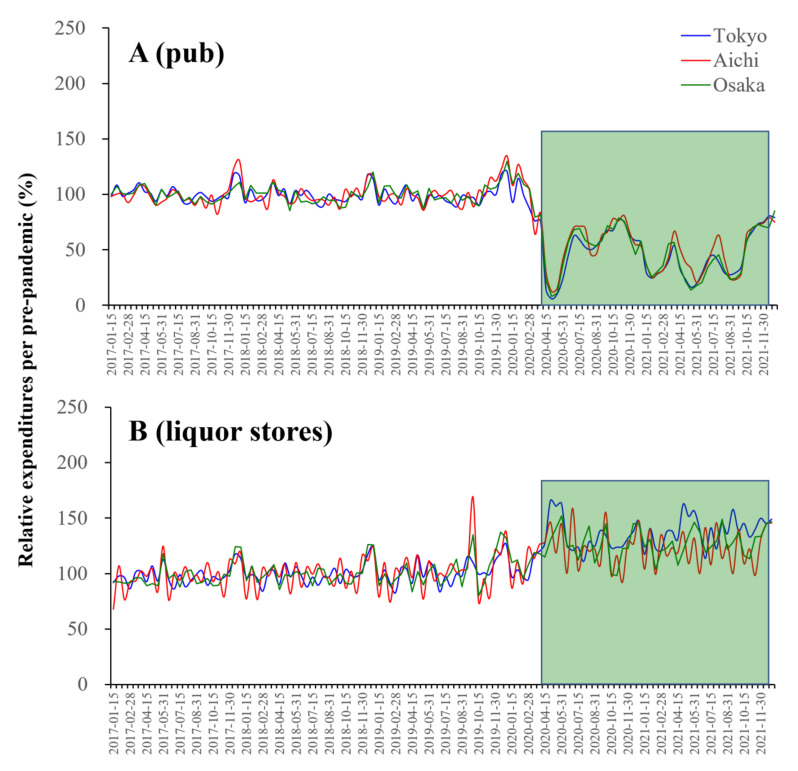
Fluctuations of bimonthly personal expenditures at pubs (**A**) and visitor numbers to pubs (**A**) and liquor stores (**B**) in Tokyo (blue line), Aichi (red line) and Osaka (green line) between January 2017 and December 2021 in Japan. Ordinates indicate the relative bimonthly personal expenditures per the average before the pandemic (January 2017–December 2019) (%). Light green columns indicate the periods of the COVID-19 pandemic in Japan.

The present study identified that an individual mobilisations with pub expenditure is one of the key players causing COVID-19 expansion [38]. Several public health and psychiatric reports depicted concerns that the prolongation of the pandemic might increase alcohol use disorder. Furthermore, alcohol consumption is considered to be a biological and sociopsychological deteriorating prognostic factor for COVID-19 [39,40]. Especially, alcohol consumption tended to elicit behaviour that promotes lower compliance to COVID-19 preventive measures [39]. Therefore, the present study determined the causality between bimonthly NCCC and expenditures at pubs and liquor stores in Tokyo, Aichi, and Osaka. Similar to the visitor numbers to pubs, expenditures to pubs during the pandemic decreased compared to the pre-pandemic levels, whereas conversely, expenditures at liquor stores increased (Figure 9). Granger causality could not detect the causality of the bimonthly NCCC from the bimonthly expenditures at pubs or liquor stores in Tokyo, Aichi, or Osaka (data not shown).

## 4. Discussion

**Figure 10:**
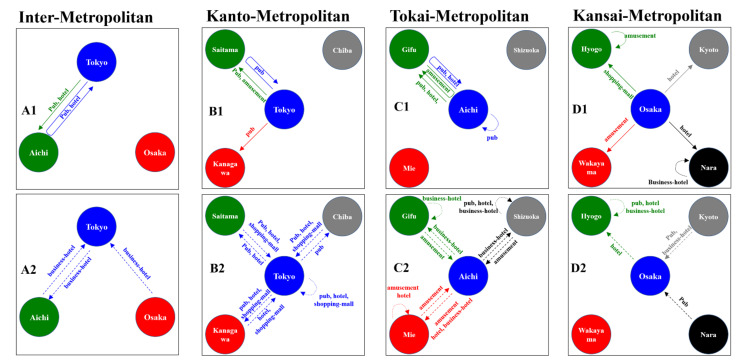
The bidirectional causalities between the numbers of newly confirmed COVID-19 cases and visitor numbers of six consumption motives (pubs, amusement places, department stores, shopping malls, hotels, and business hotels) in the three centre prefectures of the Kanto (Tokyo: blue), Tokai (Aichi: green) and Kansai (Osaka: red) metropolitan regions (**A**). The bidirectional causalities between numbers of newly confirmed COVID-19 cases and numbers of visitors of six consumption in the Kanto, Tokai, and Kansai metropolitan regions (respective in (**B**–**D**). Upper panels (**A1**,**B1**,**C1**,**D1**) indicate the impacts of individual mobilisations on the COVID-19 expanding. Lower panels (**A2**,**B2**,**C2**,**D2**) indicate the impacts of the COVID-19 expanding on individual mobilisations. The solid arrows indicate the mobilisation that is causally related to the increase in the confirmed COVID-19 cases (the arrow’s colour indicates the prefecture of increased COVID-19 cases). The blue U-type arrow indicates that increasing the visitor numbers from Tokyo to Aichi results in increasing confirmed COVID-19 cases in Tokyo. The dashed arrows indicate the decreasing visitor numbers due to the increasing confirmed COVID-19 cases (the arrow’s colour indicates the prefecture of increased COVID-19 cases).

The present study identified the inter- and intra-regional bidirectional causalities between the COVID-19 spread and individual mobilisation with consumption motives across prefectural borders in the three major metropolitan regions (Kanto, Tokai, and Kansai) in Japan. The schematic temporal relations between the bimonthly NCCC and the visitor numbers with six consumption motives are represented in Figure 10.

Using the NCCC as the sole parameter to analyse the bidirectional causalities in the three metropolitan regions, the spread of COVID-19 in Tokyo (the centre prefecture in Kanto) to both Osaka (the centre prefecture in Kansai) and Aichi (the centre prefecture in Tokai) were detected, whereas the causalities of the reversed expansion of COVID-19 from Osaka and Aichi to Tokyo could not be detected. Furthermore, the causality between Osaka and Aichi could also not be detected. These results suggested that the spread of COVID-19 in Tokyo, as a national-level epicentre, played an important role in the expansion of COVID-19 cases throughout Japan.

Using both the NCCC and individual mobilisations with six consumption motives (pubs, amusement places, department stores, shopping malls, hotels, and business hotels) as parameters, individual mobilisation from Tokyo to Aichi with consumption in pubs and staying at hotels contributed to the spread of COVID-19 in both prefectures. In contrast, individual mobilisations, such as staying at a business hotel between Tokyo and Aichi, were reduced by the spread of COVID-19 in Tokyo (increasing NCCC in Tokyo). These results imply that an individual’s mobilisation between Tokyo and Aichi for work/business purposes, with staying at a business hotel, was strictly regulated by the corresponding organisations based on the COVID-19 status; however, the individual mobilisation from Tokyo to a pub at Aichi for personal recreation motives are implicated in the spread of COVID-19 in both prefectures. Consequently, this mobilisation possibly contributed to the spread of COVID-19 in both the Kanto and Tokai metropolitan regions via similar pattern mobilisations. Several studies reported that asymptomatic and/or presymptomatic super-spreaders provide the spread of COVID-19 at pubs [12]. Therefore, the asymptomatic and/or presymptomatic super-spreaders residing in Tokyo possibly led to the spread of COVID-19 in both prefectures via pub visits. Additionally, naïve visitors from Tokyo who were infected with SARS-CoV-2 at pubs in Aichi possibly contributed to the spread of COVID-19 after returning to Tokyo. A similar pattern of the spread of the virus was observed in the Kanto and Tokai metropolitan regions when people crossed borders from the centre prefectures (Tokyo and Aichi) to other prefectures (Saitama and Gifu) to visit pubs.

The present study identified that an individual’s mobilisation with pub expenditure is one of the key players for COVID-19 expansion. Many public health and psychiatric reports are concerned that prolonging the pandemic might increase alcohol use disorder [38]. Furthermore, alcohol consumption is considered to be a biological and sociopsychological deteriorating prognostic factor for COVID-19 [39,40]. Especially, alcohol consumption tended to elicit behaviour that promotes lower compliance to COVID-19 preventive measures [39]. A recent meta-analysis also reported that slightly more individuals reported a decrease than an increase in their alcohol usage during the pandemic [41]. Decreases were also reported more often than increases in drinking frequency, quantity consumed, and heavy episodic drinking. However, the high-level drinking behaviour of individuals with alcohol use disorder was observed to have solidified/intensified [41]. In the present study, the personal expenditures at pubs decreased, and increased expenditure at liquor stores was observed during the pandemic compared to before the pandemic. Thus, major individuals who were drinking at pubs (out-of-homes) switched to drinking at home. These findings support the validity of continuously taken government measures to suppress the number of pub visitors; however, the existing restriction measures may have been insufficient, as observed in both the Kanto and Tokai metropolitan regions. Indeed, a lower number of pub visitors was observed due to the increase in NCCC in Tokyo in the Kanto metropolitan region, whereas the same decreasing trend could not be detected in the Tokai metropolitan region. Therefore, the discrepancy associated with pubs in the Kanto and Tokai metropolitan regions indicates that to prevent the spread of COVID-19, in addition to the national guidelines, the development and implementation of measures per region that are scientific and evidence-based is essential. The study design in the present study could not identify the sociopsychological characteristics of individuals who, despite social/governmental enlightenments, could not transform their alcohol consumption behaviours [42]. It is easily speculated that the multiple factors associated with individuals’ employment and living circumstances, including where and how they drank, are the target. Notably, individuals who were working remotely were more likely to increase their alcohol consumption behaviours compared to those working in person [43,44]. We should also pay attention to the fact that individuals with alcohol use disorders are solidifying/enhancing their drinking behaviour [41].

Failing to detect the specific features of mediating individual mobilisation for the spread of COVID-19 from Tokyo to Osaka suggests that motives exist other than the six considered in this study. Therefore, the causal individual mobilisation pattern for the spread of COVID-19 is probably not similar between Tokyo-Aichi and Tokyo-Osaka. Tokyo is speculated as the epicentre of the first, second, third, and fifth waves of the pandemic; meanwhile, Osaka was speculated as the epicentre of the fourth pandemic wave. In July 2021, the R1 and Alpha variants of COVID-19 spread across the Kanto and Kansai metropolitan regions, respectively; however, the Alpha variant from Osaka became predominant, resulting in a widespread infection throughout Japan [45]. Taken together, the impact of the fourth wave possibly hindered the detection of the bidirectional causalities between Tokyo and Osaka. Therefore, to assess the detailed individual causal mobilisation patterns for the spread of COVID-19 in Tokyo and Osaka, further studies that include the pandemic waves as a confounding factor, as well as considering other individual mobilisation motives, should be explored.

The causal individual mobilisation patterns for the spread of COVID-19 in the Kansai metropolitan region are relatively different from those in the Kanto or Tokai metropolitan regions. The spread of COVID-19 from Osaka to other prefectures in the Kansai metropolitan region was observed, but not from other prefectures to Osaka. Furthermore, these causal mobilisations from Osaka to other prefectures differed based on the characteristics of each prefecture. It is easy to interpret that most of the individual mobilisation for a hotel stay in Kyoto and Nara was tourism since both prefectures are ancient capitals designated as World Cultural Heritage sites by UNESCO. The individual causal mobilisations from Osaka to Hyogo and Wakayama for shopping malls and amusement place visits that contributed to the spread of COVID-19 can be attributed to the luxury shopping malls and leisure facilities, which are the respective major industries in Hyogo and Wakayama. Therefore, the causalities between the spread of COVID-19 and individual mobilisation with consumption motives across prefectural borders in the Kansai metropolitan region could easily be traced due to the nature of the industrialisation of the prefectures in this region compared with Kanto or Tokai. The restrictive measures against causal mobilisations to the hub-epicentre (Osaka) are possibly the result of comprehensive suppressions of economic activities rather than the spread of COVID-19 since the causal mobilisations are travelling to hotels, amusement places, and shopping malls. Therefore, the restriction measures for accepting visitors in the Kansai metropolitan region, focusing on the causal mobilisation for the spread of COVID-19 in the receiving prefectures, might be efficient or effective.

The suppression of individual mobilisations with consumption in pubs among bidirectional Tokyo and other prefectures was related to the increasing NCCC in the hub-centre (Tokyo) in the Kanto metropolitan region. In contrast, in both the Tokai and Kansai metropolitan regions, the bidirectional individual mobilisations between the hub-centre and other prefectures were affected by the NCCC in other prefectures and not by those in hub-centre prefectures. The causal mobilisations for the spread of COVID-19 were not in response to the increasing NCCC in the other prefectures. Therefore, the present findings, which indicate that the causal mobilisations for the spread of COVID-19 differed between the national-level and regional hub-epicentres, highlight the importance of enlightening regional individuals regarding the science-based causal mobilisations for the spread of COVID-19 across prefectural borders.

The present study has several strengths and limitations. One important strength of the study is its design, which identified the temporal bidirectional causalities between the increasing COVID-19 cases and the consumption motives of individual mobilisations using a vector autoregression model. However, the present study has several limitations that must be mentioned. The JCB consumption NOW database publishes the bimonthly visitor numbers based on personal consumption, disaggregated by prefectures, but not by sex or age. Additionally, given the serial intervals, the bimonthly consumption numbers may reflect a low temporal resolution that is insufficient to derive accurate bidirectional causality. Restaurants other than pubs also serve alcohol; however, JCB consumption NOW does not provide the visitor numbers to any restaurants other than pubs. Therefore, further study of weekly or daily visitor numbers to various types of restaurants is needed to explore a more accurate causality. The causalities between individual mobilisations and the expansion of COVID-19 should not be underestimated since it is well known that the capacity for diagnostic examination of COVID-19 was insufficient in Japan [34,35].

## 5. Conclusions

The present study identified the temporally bidirectional causalities between the numbers of newly confirmed COVID-19 cases and individual mobilisation with consumption motives. The increasing specific individual mobilisations with consumption motives from an epicentre/hub-epicentre to other prefectures before the expansion of COVID-19 was observed. Additionally, various individual mobilisations with consumption motives, which did or did not contribute to the COVID-19 spread, were also affected by the expansion of COVID-19. These bidirectional causalities between individual mobilisations and the COVID-19 expansion were complicated, possibly in a regional-dependent manner. The individual mobilisation with consumption in pubs from Tokyo to Aichi played an important role in the spread of COVID-19 in both the Kanto and Tokai metropolitan regions. These findings support the validity of continuous government measures to suppress engagement in drinking activities. In the Kanto metropolitan region, the number of pub visitors was reduced due to the increasing NCCC in the hub-epicentre (Tokyo); however, the number of pub visitors in the Tokai metropolitan region was unaffected by the spread of COVID-19 in the hub-epicentre (Aichi). Despite these efforts, sufficient prevention of expanding cases of COVID-19 in Kanto and Tokai still required the implementation of more efficient/effective restrictive measures. Meanwhile, in the Kansai metropolitan region, visitors from the hub-epicentre (Osaka) to other prefectures were positively related to the expanding cases of COVID-19 in this metropolitan region, but the causal individual mobilisation motives were dependent on the industrial nature of each prefecture. These findings suggest the importance of scientific-based evidence that evaluates the regional differences in the causal mobilisation that result in widespread COVID-19. Thus, an awareness of the impact of the identified regional causal mobilisation actions that contribute to the spread of the virus should be strengthened, and appropriate lifestyle adjustments should be implemented to prevent future pandemics.

## Data Availability

Raw data of prefectural populations are publicly available to any persons via Japanese national databases, and the Regional Statistics Database (RSD) (https://www.e-stat.go.jp/en/statistics/00200241, accessed on 1 July 2022) of the System of Social and Demographic Statistics of the Statistics Bureau of the Ministry of Internal Affairs and Communications (SBMIAC). Raw data of NCCC are also publicly available to any persons of the Database of the National Institute of Infectious Diseases (https://www.niid.go.jp/niid/ja/calendar.html, accessed on 1 July 2022) and the Sapporo Medical University School of Medicine (https://web.sapmed.ac.jp/canmol/coronavirus/japan.html, accessed on 1 July 2022). Raw data on personal consumption were purchased from “JCB Consumption NOW” (Nowcast, Tokyo, Japan) (https://www.jcbconsumptionnow.com/en; accessed on 1 May 2022).

## Supplementary Materials

The following supporting information can be downloaded at: https://www.mdpi.com/article/10.3390/ijerph19159070/s1.
