# Supplementary material for: Bidirectional Causality between Spreading COVID-19 and Individual Mobilisation with Consumption Motives across Prefectural Borders in Japan
Source: Int J Environ Res Public Health. 2022 Jul 25;19(15):9070. doi: 10.3390/ijerph19159070 (PMC9332297; doi:10.3390/ijerph19159070)
Supplement: Supplementary file 1 [file ijerph-19-09070-s001.zip › ijerph-1804240-supplementary.pdf]

Supplementary Data List

Supplementary Figure S1: Temporally bidirectional causalities of from bimonthly NCCC to visitor numbers among centre prefectures of three (Kanto, Tokai and Kansai) metropolitan regions (p2)

Supplementary Figure S2: Temporally bidirectional causalities of from bimonthly NCCC in Tokyo to numbers of pubs visitors among prefectures in Kanto metropolitan region (p3)

Supplementary Figure S3: Temporally bidirectional causalities of from bimonthly NCCC in Tokyo to numbers of hotels visitors among prefectures in Kanto metropolitan region (p4)

Supplementary Figure S4: Temporally bidirectional causalities of from bimonthly NCCC in Tokyo to numbers of shopping malls visitors among prefectures in Kanto metropolitan region (p5)

Supplementary Figure S5: Temporally bidirectional causalities of from bimonthly NCCC in prefectures in Tokai metropolitan region to visitor numbers of pubs and amusement places among prefectures in Tokai metropolitan region (p6)

Supplementary Figure S6: Temporally bidirectional causalities of from bimonthly NCCC in prefectures in Tokai metropolitan region to visitor numbers of hotels and business hotels among prefectures in Tokai metropolitan region (p7)

Supplementary Figure S7: Temporally bidirectional causalities of from bimonthly NCCC in prefectures in Kansai metropolitan region to visitor numbers of pubs, hotels and business hotels among prefectures in Kansai metropolitan region (p8)

Supplementary Figure S1:

Temporally bidirectional causalities of from bimonthly NCCC to visitor numbers among centre prefectures of three (Kanto, Tokai and Kansai) metropolitan regions.

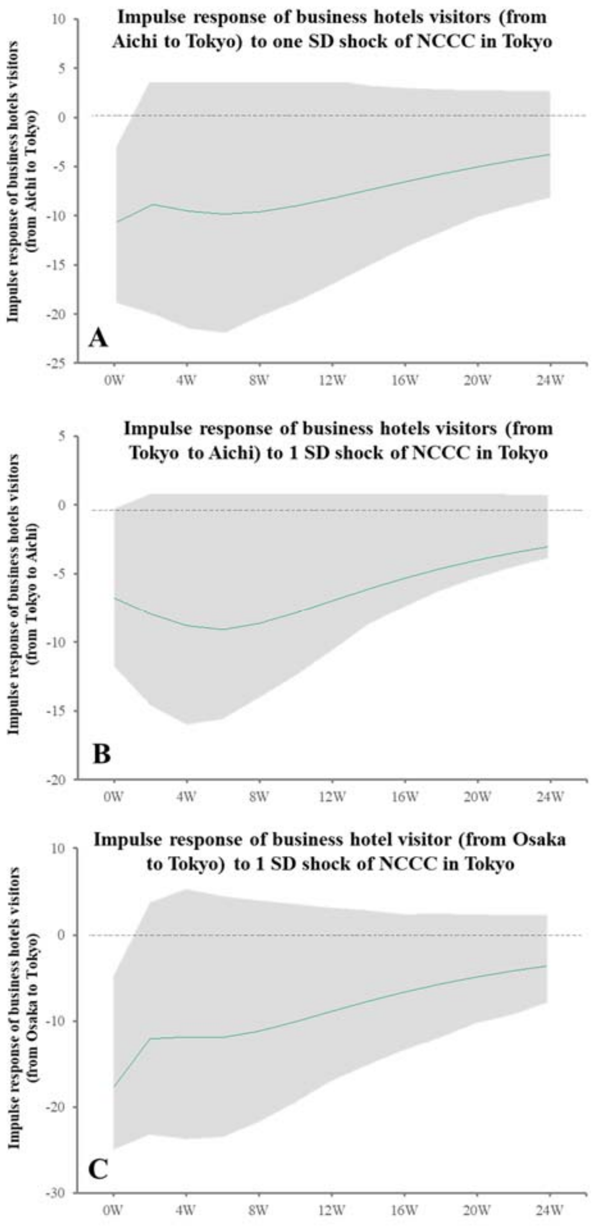

Panels A -C indicate the impulse responses of bimonthly business hotels visitor (from Aichi to Tokyo, from Tokyo to Aichi and from Osaka to Tokyo) to bimonthly NCCC in Tokyo, Aichi and Osaka, respectively. Green lines and gray regions indicate the mean± 95% confidence interval (CI) of responses.

Supplementary Figure S2:

Temporally bidirectional causalities of from bimonthly NCCC in Tokyo to numbers of pubs visitors among prefectures in Kanto metropolitan region.

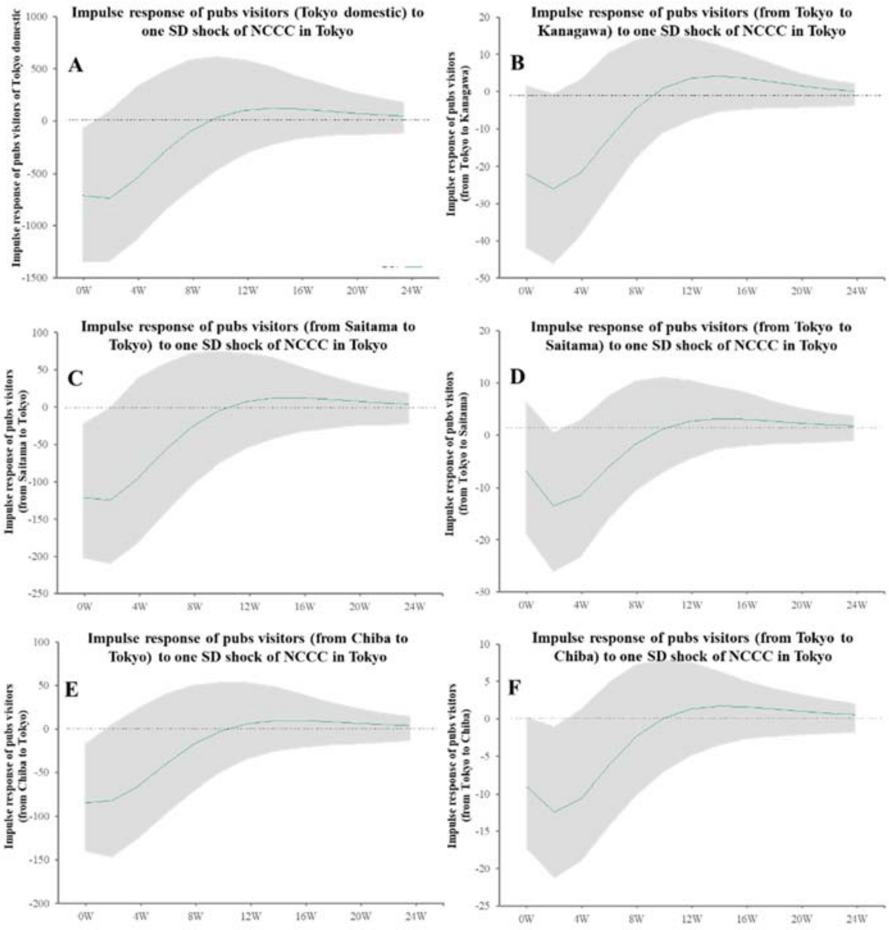

Panels indicate the impulse responses of bimonthly pubs visitors to bimonthly NCCC in Tokyo. Green lines and gray regions indicate the mean± 95%CI of responses.

Supplementary Figure S3:

Temporally bidirectional causalities of from bimonthly NCCC in Tokyo to numbers of hotels visitors among prefectures in Kanto metropolitan region.

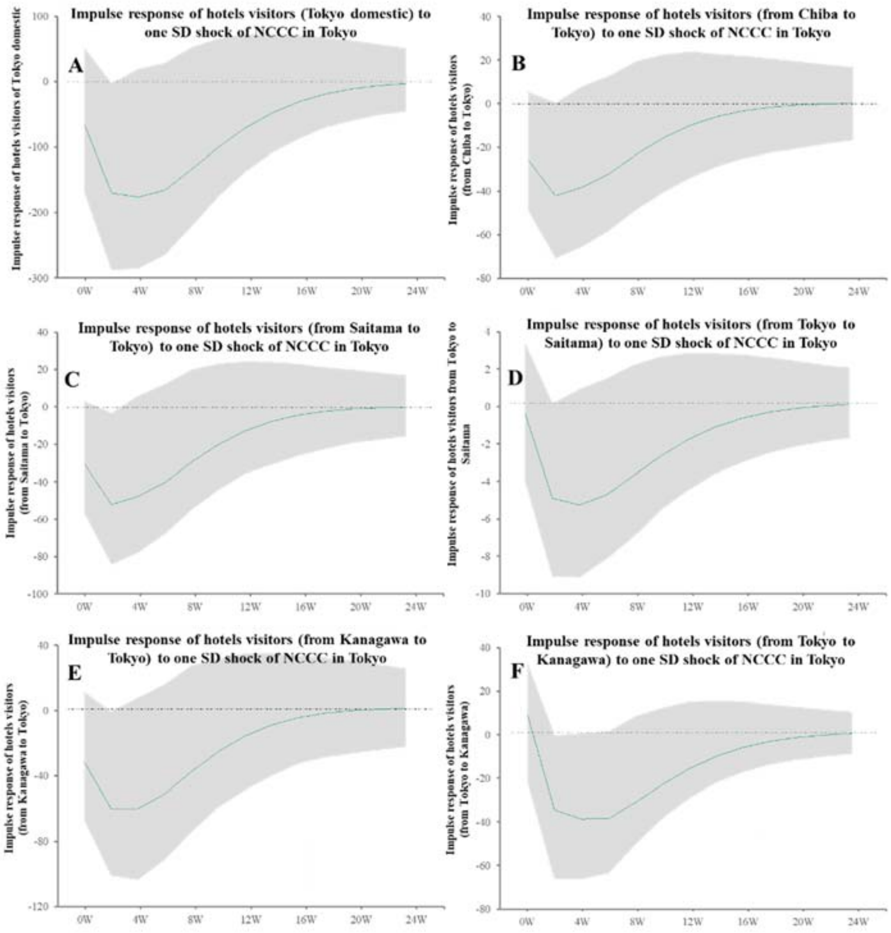

Panels indicate the impulse responses of bimonthly hotels visitors to bimonthly NCCC in Tokyo. Green lines and gray regions indicate the mean± 95%CI of responses.

Supplementary Figure S4:

Temporally bidirectional causalities of from bimonthly NCCC in Tokyo to numbers of shopping malls visitors among prefectures in Kanto metropolitan region.

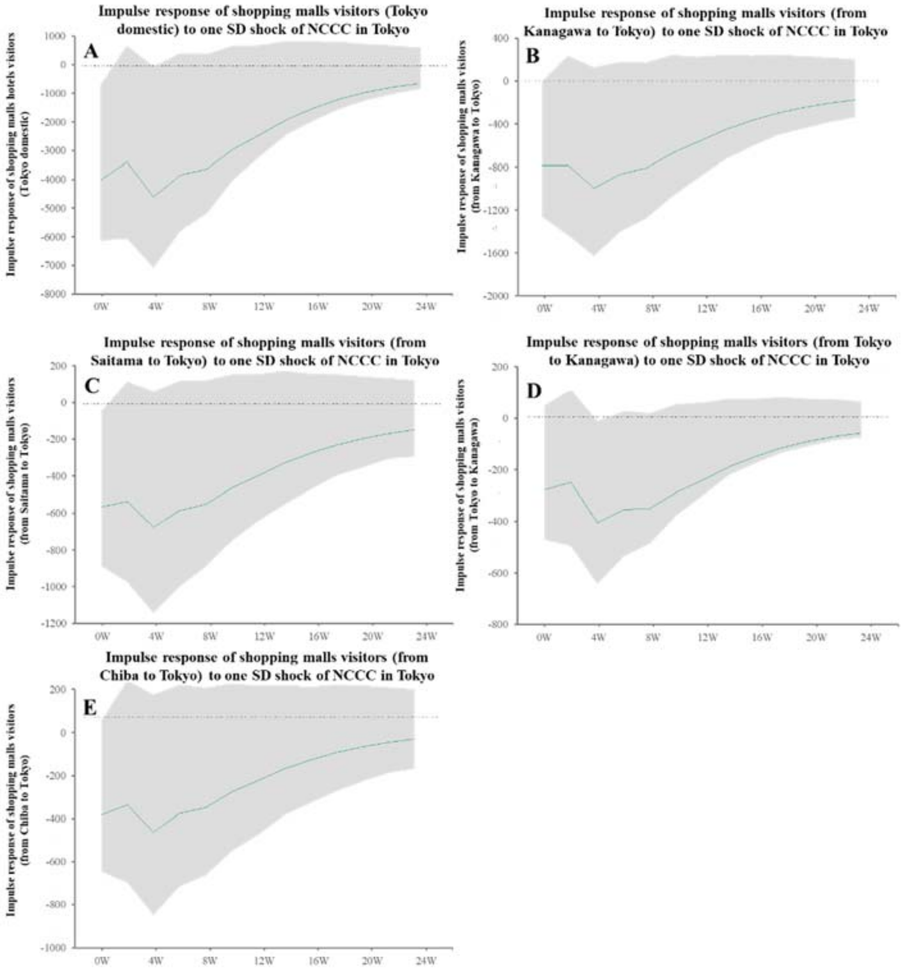

Panels indicate the impulse responses of bimonthly shopping malls visitors to bimonthly NCCC in Tokyo. Green lines and gray regions indicate the mean± 95%CI of responses.

Supplementary Figure S5:

Temporally bidirectional causalities of from bimonthly NCCC in prefectures in Tokai metropolitan region to visitor numbers of pubs and amusement places among prefectures in Tokai metropolitan region.

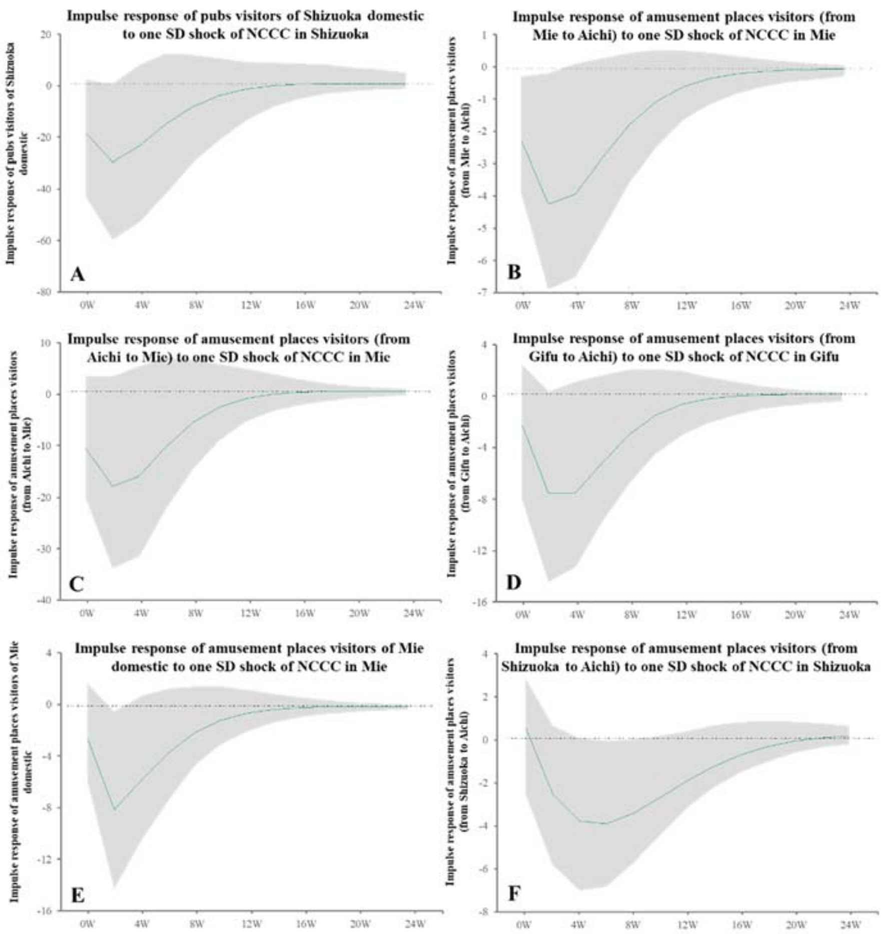

Panels indicate the impulse responses of bimonthly visitor numbers of pubs and amusement places to bimonthly NCCC in prefectures in Tokai metropolitan region. Green lines and gray regions indicate the mean± 95%CI of responses.

Supplementary Figure S6:

Temporally bidirectional causalities of from bimonthly NCCC in prefectures in Tokai metropolitan region to visitor numbers of hotels and business hotels among prefectures in Tokai metropolitan region.

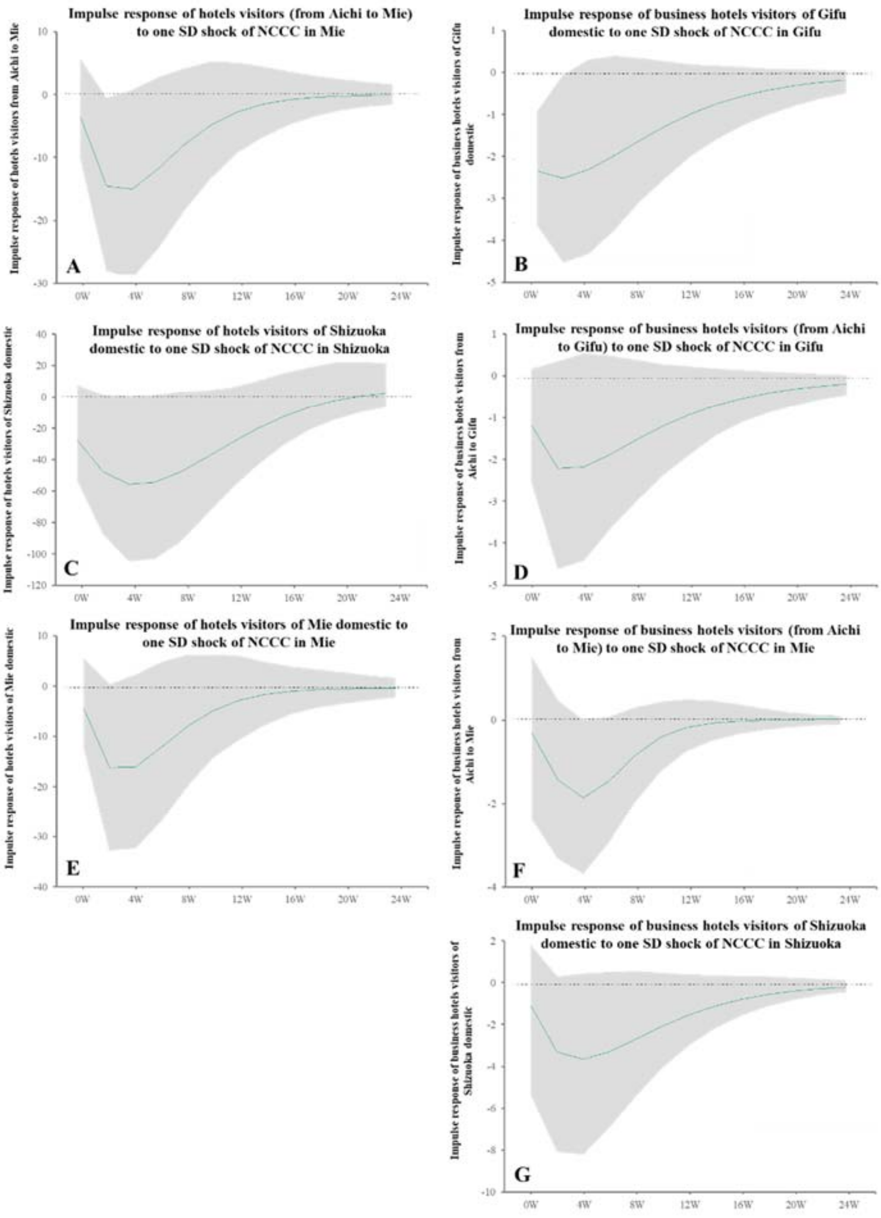

Panels indicate the impulse responses of bimonthly visitor numbers of hotels and business hotels to bimonthly NCCC in prefectures in Tokai metropolitan region. Green lines and gray regions indicate the mean± 95%CI of responses.

Supplementary Figure S7:

Temporally bidirectional causalities of from bimonthly NCCC in prefectures in Kansai metropolitan region to visitor numbers of pubs, hotels and business hotels among prefectures in Kansai metropolitan region.

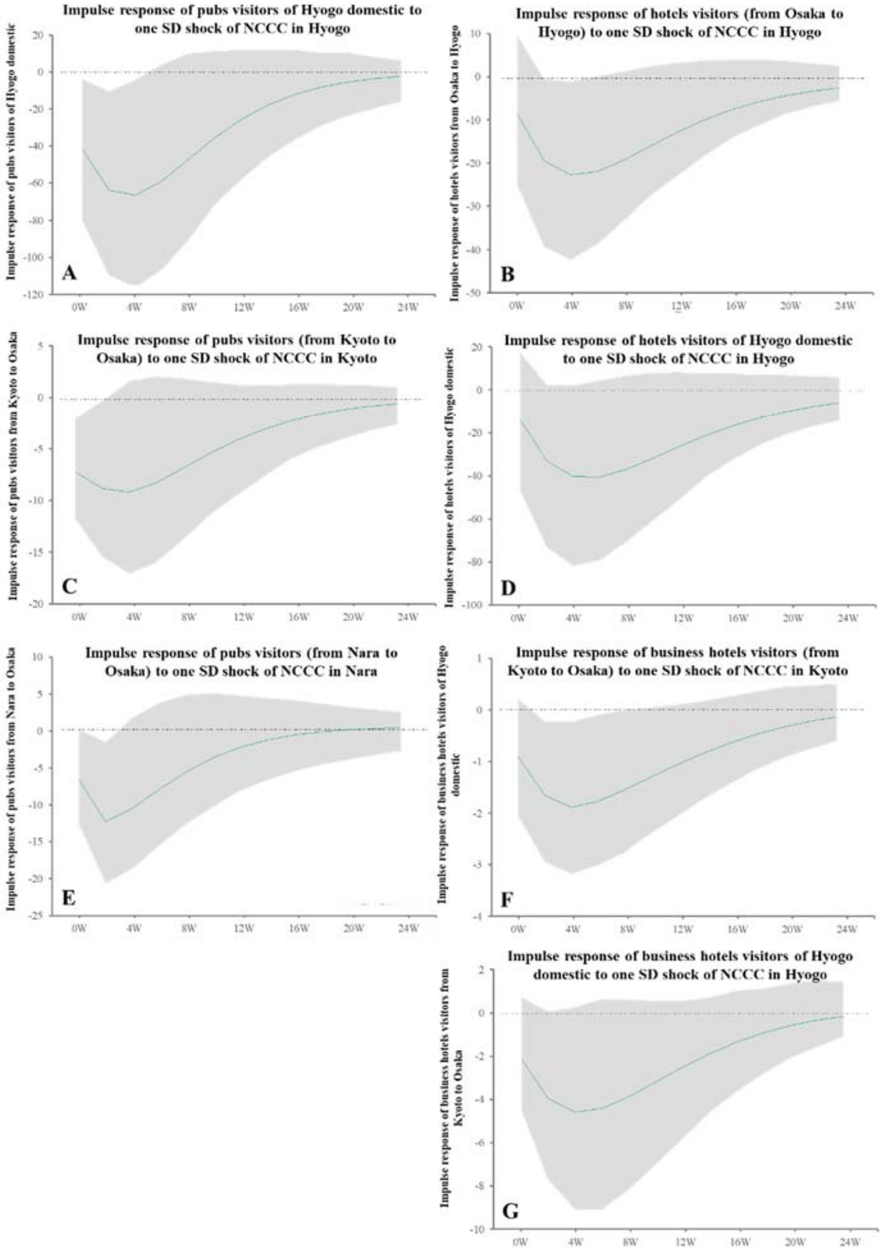

Panels indicate the impulse responses of bimonthly visitor numbers of pubs, hotels and business hotels to bimonthly NCCC in prefectures in Kansai metropolitan region. Green lines and gray regions indicate the mean± 95%CI of responses.
